# SitesIdentify: a protein functional site prediction tool

**DOI:** 10.1186/1471-2105-10-379

**Published:** 2009-11-18

**Authors:** Tracey Bray, Pedro Chan, Salim Bougouffa, Richard Greaves, Andrew J Doig, Jim Warwicker

**Affiliations:** 1 Faculty of Life Sciences, The University of Manchester, Michael Smith Building, Oxford Road, Manchester M13 9PT, UK; 2 Manchester Interdisciplinary Biocentre, The University of Manchester, 131 Princess Street, Manchester M1 7DN, UK

## Abstract

**Background:**

The rate of protein structures being deposited in the Protein Data Bank surpasses the capacity to experimentally characterise them and therefore computational methods to analyse these structures have become increasingly important. Identifying the region of the protein most likely to be involved in function is useful in order to gain information about its potential role. There are many available approaches to predict functional site, but many are not made available via a publicly-accessible application.

**Results:**

Here we present a functional site prediction tool (SitesIdentify), based on combining sequence conservation information with geometry-based cleft identification, that is freely available via a web-server. We have shown that SitesIdentify compares favourably to other functional site prediction tools in a comparison of seven methods on a non-redundant set of 237 enzymes with annotated active sites.

**Conclusion:**

SitesIdentify is able to produce comparable accuracy in predicting functional sites to its closest available counterpart, but in addition achieves improved accuracy for proteins with few characterised homologues. SitesIdentify is available via a webserver at http://www.manchester.ac.uk/bioinformatics/sitesidentify/

## Background

Efforts, primarily by structural genomics groups, have provided a rapidly growing number of protein structures with little or no functional annotation. This has caused new interest in the relationship between structure and function and has increased focus on ways to elucidate a protein's function from its structure rather than solely from sequence. In order to investigate the role of a protein using its structure, it is useful to be able to identify the portion of the protein that is most closely involved with its function. In the case of enzymes this is its active site, whilst non-enzymes have functionally important regions that are involved in ligand-binding or protein-protein interactions.

There are currently several computational approaches that predict functional sites which use either structural or sequence information. The most widely used methods rely on sequence information in order to predict functionally important residues, due to the greater availability of sequence data as opposed to structural data for uncharacterised proteins. Sequence based methods mainly centre around the concept of functionally important residues being more highly conserved through evolution and identify the most conserved residues by comparing positions in a multiple sequence alignment with homologous proteins. Some methods use only sequence conservation information in making predictions [1,2], whilst others also include additional computed sequence features [3], or structural properties predicted from sequence such as predicted secondary structure and solvent accessible surface area [4,5], particularly in order to distinguish between residues conserved for function and those conserved for structure [6,7]. Many methods focus on predicting catalytic residues in enzyme active sites, but measures of sequence conservation have also been successfully used to predict residues in contact with a ligand [1,5,8] or in contact with other proteins, although sequence conservation has been shown to perform less well as a predictive feature in the latter cases [1,9].

Whilst there are a large number of sequence-based methods available, there are also a growing number of methods that predict functional sites based on structural information. These methods fall into two main categories: those that identify structural similarities and transfer annotation from a protein with a known functional site and those that predict functional sites by non-homology related structural features such as geometrical or electrostatic properties [5,10,11].

There are many resources that store structural and sequence information about proteins with known active sites, such as PdbFun [12], CSA [13], PDBSite [14] and ProSite [15]. A protein of unknown active site location can be compared to these resources (CSS [16] scans the CSA and PDBSiteScan [17] scans PDBSite), or to databases derived specifically for the prediction method, to identify any structural similarities with known active sites [18-25]. While these methods often produce accurate results, they assume the existence of a functionally annotated homologue of similar active site structure in their respective databases. As one of the aims of structural genomics initiatives is to obtain structures for proteins that occupy remote fold space, these methods may be of limited use for such proteins.

In this situation, *ab initio *methods that do not rely on the existence of a functionally characterised homologue may be of more value. A wide range of structural properties have been used, showing that the relationship between a protein's structure and its function is affected by many structural characteristics. A study of catalytic residues and their properties [26] showed that they are likely to exist in regions of the protein that are not in helix or sheet secondary structure, have a higher propensity to be a charged residue and exhibit lower B-values than non-catalytic residues. A number of methods have used these characteristics to predict residues involved in catalysis [27,28]. Bartlett et al. noted that catalytic residues tend to line the surface of large surface clefts, yet remain relatively buried within the protein geometry. It was also observed in a study of 67 single-chain enzymes that 83% of enzyme active sites are found in the largest surface cleft [29], resulting in methods to predict active sites by finding surface clefts [30,31].

Previous work by this group [32] attempted to identify functional sites by locating peak electrostatic potentials near to the surface of a protein resulting from the interaction of charged residues that are under electrostatic strain. The greatest functional site prediction accuracy, however, was obtained by applying a uniform charge weighting across the protein rather than using actual charges. This uniform charge weighting essentially acts as a cleft-finding algorithm and will predict the most buried surface cleft. This gave a prediction accuracy of 77%, where a successful prediction is when the peak potential was within 5% of the protein surface from the real active site centre.

Other studies have successfully used electrostatics calculations to predict active site and ligand-binding site residues [33-37]. Elcock identified residues that had destabilizing effects on the stability of the protein using continuum electrostatics methods and found that these correlated with residues involved in protein functionality [33]. This method, however, was not tested on a large experimentally annotated dataset and so it is hard to interpret the degree of accuracy it achieved. Another approach predicts enzyme active sites by identifying residues with unusually-shaped titration curves [35,38] as well as predicting enzyme function [39]. Other chemistry-based approaches, such as identifying residues that are unusually hydrophobic for their position in a structure have also been successful [40].

Other *ab initio *methods use the degree of connectivity of residues to predict those involved in function. A number of methods assess the closeness centrality of residues [41-43], whilst one study found that catalytic residues are more likely to exist in close proximity to the molecular centroid [44].

Perhaps the best accuracies can be achieved by combining structural approaches and sequence conservation. Residues may be evolutionarily conserved due to structural as well as functional constraints and a number of studies have attempted to distinguish these two factors by considering the degree of conservation and the residue's structural environment [6,45]. Mapping the degree of evolutionary conservation onto the structure is useful in identifying clusters of conserved residues in the structure that may indicate a functional site [46,47]. Combining the types of structural information used in *ab initio *structural methods with sequence conservation can be effective [10,11,34,48,49].

Despite the success of the large number of varied approaches, only a relatively small subset of these methods are currently available either via a software package or a web-server. Tools report various levels of accuracy that are difficult for a user to compare due to their separate test datasets, outputs and reporting methods. Here we present a user-friendly functional site prediction tool, SitesIdentify, based on previously published work by this group [10,32]. This is made publicly available via a web-server [50], and is compared to other accessible tools in a comparison of performance on a common dataset.

## Implementation

### Functional Site Prediction Methods

SitesIdentify can predict functional site location by two separate approaches, which have been described in more detail in previous publications [10,32]. In brief, the first method [32] places a 2Å grid over the protein structure and applies a uniform charge to each non-hydrogen atom. The electrostatic potential is calculated using Finite Difference Poisson-Boltzmann calculations with no dielectric boundary. The peak potential is predicted as the centroid of the functional site.

The second method [10] combines the electrostatics method used above with sequence conservation information. Close homologues are found by running the sequence through PSI-BLAST with an E value cut-off of 1e-20. A normalised conservation score is calculated for each residue based on the amino acid and stereochemical diversity and the gap occurrence at that position, *C*(*x*) = (1-t(*x*))^α^(1-*r*(*x*))^β^(1-*g*(*x*))^γ^, where *t *is the normalised symbol diversity, *r *is the normalised stereochemical diversity (based on the BLOSUM-62 matrix) and *g *the gap cost. Each of these terms are weighted by integral values ranging between 0 and 5 (α, β and γ), the values for which are defined as those giving the best predictive performance in the original publication [10]. The peak potential is then calculated in the same way as the first method, but now with a single central atom in each amino acid weighted with the conservation scores.

### SitesIdentify Workflow

Upon submission of a job, SitesIdentify starts a number of programs depending on which method the user requested. If the conservation approach is selected, the in-house Conserved Residue Colouring program(CRC) is run first, which identifies homologues by running the sequence contained in the SEQRES records in the PDB file through PSI-BLAST [51]. PSI-BLAST is run for one iteration (in default settings) on the non-redundant database with an E-value cut-off for inclusion of sequences of 1e-20. A profile file containing the conservation scores for each residue is produced. SitesIdentify uses the conservation scores as charge weightings on a single atom for each amino acid (C_β _or C_α _for glycine), and calculates the location of the peak potential as described above [10]. If no homologue can be identified for a protein using CRC then the method automatically switches to only charge-based calculations. If the conservation method is not selected then the CRC program is omitted and the location of the peak potential is calculated using the uniform charge-weighting method [32]. A sphere of user-supplied radius is drawn around the predicted centroid coordinates and residues are selected that have at least one atom within that sphere and also exhibit more than 5Å^2 ^of solvent-accessible surface area (SASA) as calculated using the Lee and Richards method [52]. This list of residues represents the predicted functional site, which is given in the results as a text list and also highlighted on the PDB structure using Jmol [53].

### SitesIdentify Usage

SitesIdentify is available for use via a web browser and is freely accessible without license or an account registration. The main web page allows a user to enter either a pre-existing PDB structure ID (and whether to use the biological unit or the asymmetric unit) or upload a structure file, the radius around the predicted site to use, the method to use and an email address so that a user can be notified and emailed the results link upon job completion.

If a user has submitted their own structure file then this is validated to ensure that contains an acceptable PDB-format structure, the rules for which are given in the user guide available from the website. The file must be less than 2 MB in size and contain only text. It also must contain at least SEQRES and ATOM records and be spaced exactly as the standard PDB format. If the user-supplied information is invalid (non-existent PDB ID or invalid email address) then the job is not initialized and the user informed of the incorrect information via the browser. Upon successful completion of a job the web-server directs the user to the results page and also sends an email to the user at the address specified with a link to the results page.

## Results and Discussion

### SitesIdentify Web-server

**Figure 1:**
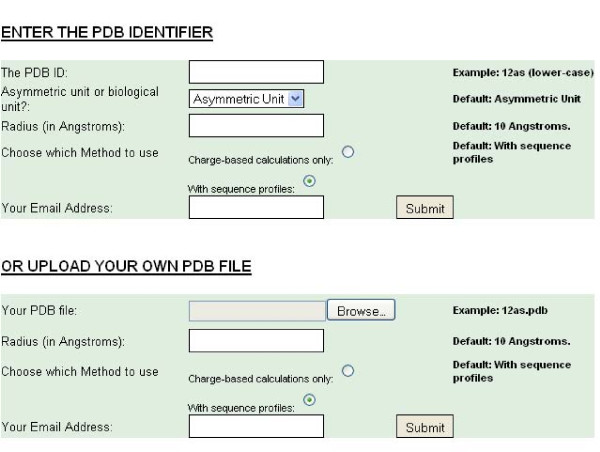
**Screenshot showing the required user input fields**. A user can either input a pre-existing PDB code and whether to use the asymmetric or biological unit structure or upload their own PDB-style structure file. All fields are compulsory.

SitesIdentify is available to run for single protein entries at http://www.manchester.ac.uk/bioinformatics/sitesidentify/ or can be downloaded to run offline for multiple proteins (Additional File 1). It requires some basic user-input via a web-browser (see Figure 1). Once this information is validated a new job is initiated. The average calculation time per protein is approximately 6 minutes when using the method including conservation information and approximately 2 minutes if only using charge-based calculations. If the protein takes longer than 45 minutes to produce results, which may occur for very large proteins, the job is terminated and the user is notified by email.

**Figure 2:**
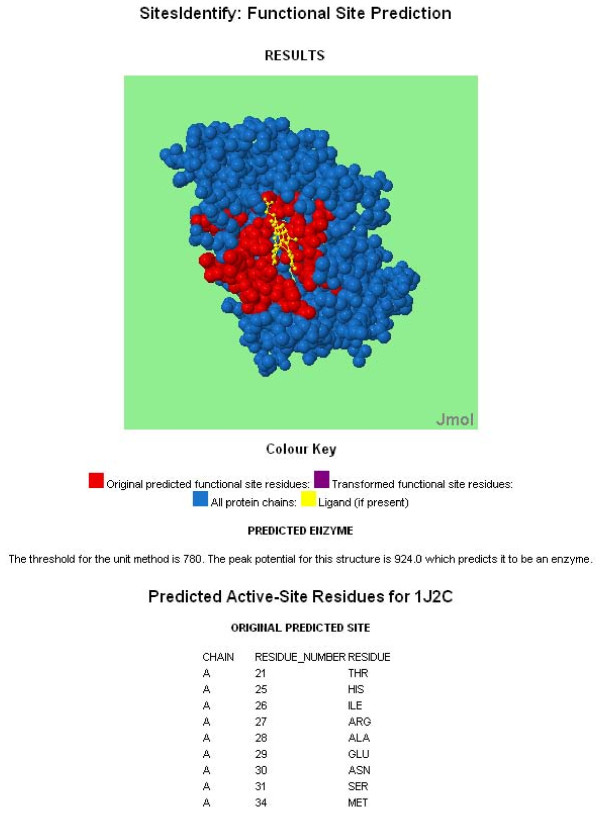
**Screenshot of an example results output for SitesIdentify**. The output for 1j2c (rat heme oxygenase-1) when submitted using the charge-based method and a 10Ǻ radius. The list of active site residues is truncated for display purposes.

Upon completion of a job an email is sent to the user at the address specified which provides a link to the results page. The results page displays a Jmol applet illustrating the protein structure with the predicted site residues highlighted, a text list of the predicted residues and a link to a text file containing the predicted residue information (see Figure 2 for an example).

The methods used in SitesIdentify can distinguish between enzyme and non-enzyme with a high degree of accuracy [32] and so an enzyme/non-enzyme prediction is also given along with the functional site prediction. Cleft size has also been used as a discriminator between enzyme and non-enzyme with enzymes more likely to exhibit large surface accessible clefts than non-enzymes [54]. Since the charge-based method essentially identifies buried clefts it is likely to perform better for enzymes than non-enzymes, although it still may be able to detect small ligand-binding pocket clefts in non-enzymes. In addition, the second SitesIdentfiy method incorporates sequence conservation information which has also been shown to useful in predicting other biologically important regions such as non-enzyme ligand binding sites [49], protein-protein interaction sites [55-57] and DNA-binding sites [58]. It is worth noting however, that a study of four non-enzyme families by Magliery et al. found that rather than binding sites being conserved, they showed a higher degree of variation than the rest of the protein [8]. This may explain why some conservation approaches report better accuracies in predicting functional sites of enzymes than non-enzymes [49,59].

It is unsurprising therefore that SitesIdentify performs better for enzymes than non-enzymes although it is still able to identify non-enzyme ligand-binding sites with comparable accuracies to other non-enzyme specific functional site tools (see Additional File 2). This tool may extend to identifying other non-enzyme functional sites such as protein-protein interactions and DNA interactions but this is, as yet, untested. It is therefore useful to the user if analysing a protein of unknown function to predict whether the structure is an enzyme or non-enzyme when choosing the method of SitesIdentify to use and interpreting its results.

**Figure 3:**
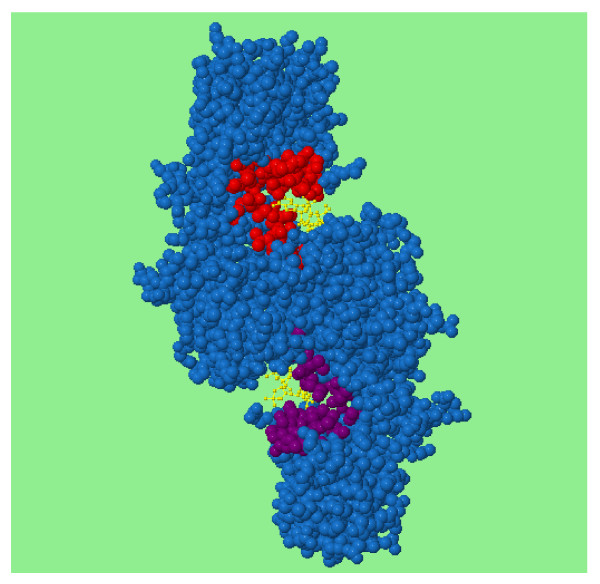
**An example of highlighted residues in an alternative predicted site**. The biological unit structure for 2af4 (phosphotransacetylase) is a homodimer and identical active sites are present on both chains. SitesIdentify identifies only one site (in red), but the annotation is transformed onto the other chain in order to identify the other active site (shown in purple).

SitesIdentify only gives a prediction for a single functional site as it makes predictions based on the single highest peak potential. In oligomeric structures, however, the same site may be present in multiple subunits and so where there is a similar site in other chains SitesIdentify identifies it as another possible site. These residues are highlighted in purple on the protein structure (see Figure 3).

Where a user inputs a pre-existing PDB ID to SitesIdentify, the option to use either the asymmetric unit or the biological unit structure is given. Where the real functional site is formed in or near subunit boundaries in the biological unit, running SitesIdentify on the asymmetric unit may fail to give the correct prediction.

**Figure 4:**
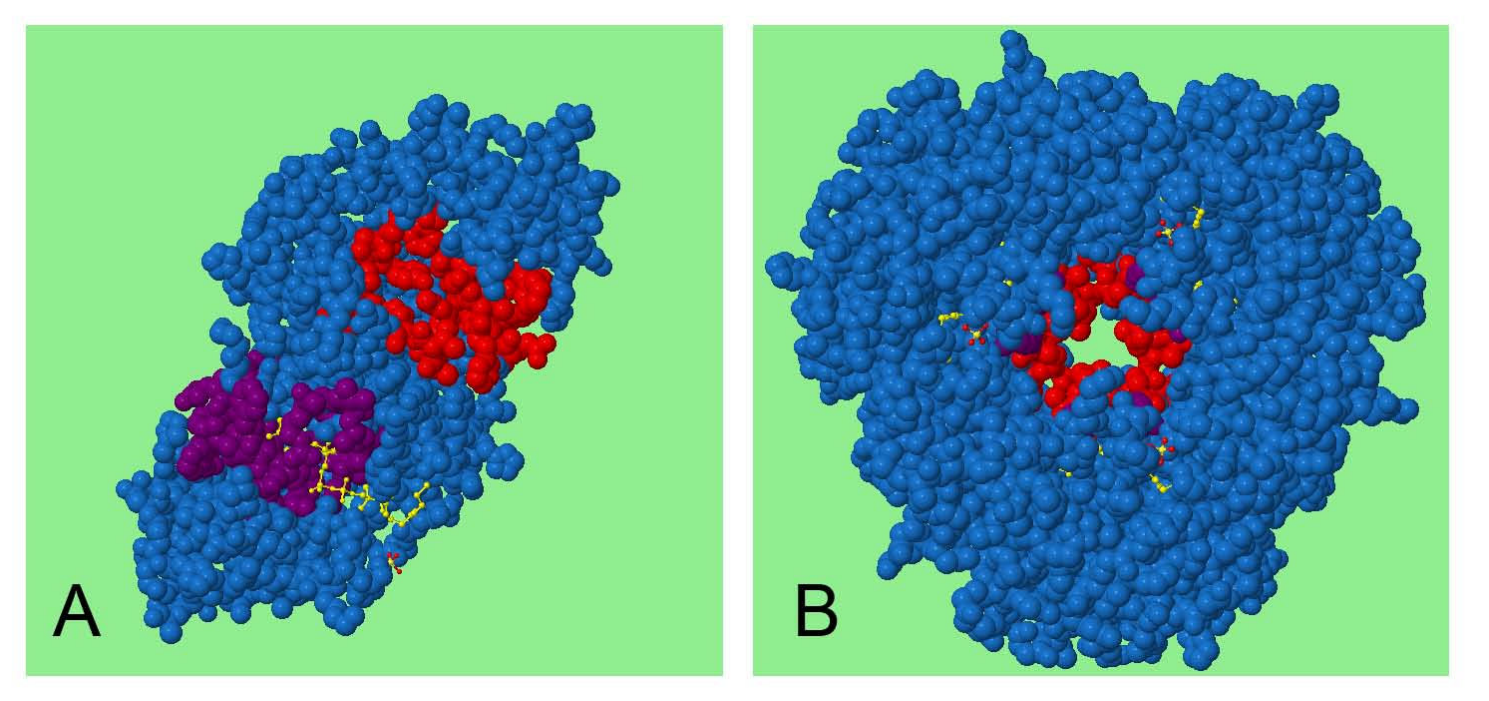
**An example of differential site prediction between asymmetric and biological unit structures**. The active site predicted for the asymmetric unit of 1b6t (phosphopantetheine adenylyltransferase) is reasonably close to the bound ligand shown in part A. The biological unit is formed by a cyclical arrangement of the asymmetric unit and when SitesIdentify is run on this structure it incorrectly identifies the central void as the enzyme active site (part B).

Some biological units, however, may give a false prediction particularly where there is an internal void formed by a cyclical arrangement of subunits. Such voids tend to be well-buried, more so than the real surface clefts, and the residues on the edges of these voids may be evolutionarily conserved in order to retain the quaternary structure. These voids are therefore sometimes incorrectly selected as predicted functional sites, and so where a biological unit has an internal void it would be useful to also run SitesIdentify on the asymmetric unit. For example, running the asymmetric unit for 1B6T through the SitesIdentify server locates the functional site in the correct location, however the site is predicted incorrectly for the biological unit as the void formed in the centre of the molecule (see Figure 4).

### Comparison to Other Applications

It is difficult to objectively compare the accuracy levels reported by the authors of the various existing functional site prediction tools as they use different datasets and report predictions differently. Some tools predict only the residues specifically involved in the protein function (e.g. catalysis) whilst others predict all residues in and around the functional site. Here, we have attempted to compare suitable methods on a common dataset of non-redundant proteins with known functional sites.

Some methods only predict enzyme active sites whilst others can identify functional residues in other types of proteins (for example PDBSiteScan and Q-SiteFinder). Enzyme active sites are the most easily defined functional sites in proteins and are the most common prediction targets for these tools; therefore the dataset we have used here contains enzymes with known catalytic residues (results for an analysis on a small set of non-enzymes is available in Additional File 2). The Catalytic Sites Atlas (CSA) [26] is a valuable resource for storing information about catalytic residues that are annotated from literature and at the time of creation of this dataset (November 2008) it contained 880 enzymes with literature-annotated catalytic residues (version 2.2.1). These were then culled for redundancy to ensure that no two structures contained an active-site domain from the same SCOP [60] superfamily (detail of this culling procedure has been reported [61]). This produced a non-redundant set of 237 enzymes for which there are annotated catalytic residues (see Additional File 3 for the list of PDB codes).

In order to be included in this analysis, a method had to adhere to the following criteria:

• The method must require no prior knowledge about the active site.

• It produces output that identifies the active site either by a coordinate location, the identities of catalytic residues or identities of residues found in the binding site.

• It produces results within a reasonable time scale. The method should return results for a test protein with 330 residues in 10 minutes or less.

• It does not simply access known annotation about the test protein.

The applications that met these criteria are listed in Table 1. Other applications that were considered but were not included in this study, along with the reason for not including them, are listed in Additional File 4. Where a method only accepts one chain from a PDB structure, the first chain is used. All predictions are run on the asymmetric unit structure.

**Table 1:** Functional site prediction tools included in the comparison analysis.

Application	Method Category	Description	Reference
**SitesIdentify**			
Uniform charge method	CF	A uniform charge weighting is applied to each Cα atom on the protein and the electrostatic potential (Finite Difference-Poisson-Boltzmann calculation with no dielectric boundary) is sampled at points on a 2Ǻ grid across the protein volume. The peak potential indicates the position of the predicted active site.	Bate and Warwicker, (2004)
Conservation method	SC, CF	As for the above method, except that the charge weightings applied across the protein are replaced with conservation weights derived from normalised sequence profile scores reflecting the amino acid diversity, the stereochemical diversity and the gap occurrence.	Greaves and Warwicker, (2005)
**Consurf**	SC	Consurf calculates the degree of evolutionary conservation for each residue in a structure and gives them an integer score from 1 to 9, with 9 being the most conserved residues. A graphical representation of the structure is then coloured according to these residue conservation scores, which allows visual identification of highly conserved patches that are predicted to be functional sites.	Landau et al. (2005)
**Crescendo**	SC	Predicts active sites by identifying clusters of residues that have higher than usual evolutionary restraint. Evolutionary constraint was identified by three measures: 1) whether there was a higher degree of evolutionary conservation than expected at a position, 2) whether environment specific substitution tables made weak predictions of the amino acid substitution patterns, and 3) residues that have spatially conserved positions when structures of proteins within the same family are superimposed.	Chelliah et al. (2004)
**FOD**	HP	The active site residues are predicted to be those with the highest hydrophobic deficiency score. This is the difference between the expected hydrophobicity and the observed hydrophobicity value for each residue. The expected hydrophobicity of a residue is determined by a residues relative position to the theoretically most hydrophobic point in the protein. The observed hydrophobicity is a combination of the hydrophobicity value of that residue and the effect on the residues position of other sidechains around it.	Brylinksi et al. (2007)
**Q-SiteFinder**	CF	Non-bonded interaction energies are calculated by placing a 3D grid over the whole protein and then evaluating the interaction energy between the protein and a methyl group at each point on the grid. The positions of the probes on the grid that gave the best interaction energies were then spatially clustered to identify groups of close probes. These clusters are then assigned a single interaction energy based on the energies of their member probes. The clusters are then ranked by their representative interaction energy and the highest ranked cluster is predicted as the active site.	Laurie and Jackson (2005)
**PDBSiteScan**	TM	PDBSiteScan takes 3D fragments of a protein structure and compares them to 3D structure fragments of known active sites. The known active sites structures are held in a collection called PDBSite that is formed from annotation in the PDB SITE field and also REMARK 800 fields. Results were discounted if they compared to annotation held for the test protein.	Ivanisenko et al. (2004)
**PASS**	CF	PASS (Putative Active Site Spheres) is essentially a geometric cleft-finding method. The shape, volume and depth of the cleft determine which clefts are predicted as active site clefts.	Brady and Stouten (2000)
**Thematics**	CP	Thematics identifies ionisable residues with unusually perturbed titrations curves. Active sites are predicted where two or more of these ionisable residues form a cluster in 3D space.	Wei et al. (2007)

A description of the seven tools used in this analysis along with a brief description of each method. Method categories are as follows: CF = cleft-finding, SC = sequence conservation, HP = hydrophobicity, TM = structural template matching, CP = chemical properties.

In order to put predictions into the same context as those given by SitesIdentify, a central PDB coordinate point is calculated for each prediction given by each method. For example, if a method only predicts catalytic residues, the central coordinate point (centroid) is defined as the geometric average of the C_β _atom (C_α _for glycine) coordinates of the catalytic residues. Similarly to the SitesIdentify output, a sphere with a 10Å radius is drawn around this centroid and residues are selected if they have at least one atom within this radius and also have a SASA of 5Å^2 ^or more. These residues are termed the standardised predicted residues.

There are three measures of accuracy used in this analysis. The first is the average percentage of annotated catalytic residues for each protein that are included in the standardised predicted residues (average absolute recall rate). Second is the average absolute recall rate for the method divided by the absolute recall rate of catalytic residues returned by the real centroid (the average relative recall rate). Third is simply the Cartesian distance from the real centroid and the predicted centroid.

It is more representative to consider the relative recall rate for each method as opposed to the absolute recall rate as for some proteins less than 100% of the annotated catalytic residues are recalled by selecting residues that have at least 5Å^2 ^SASA within a 10Å radius. It is therefore unlikely for these proteins that even a very accurate prediction would give an absolute recall rate of 100%.

**Figure 5:**
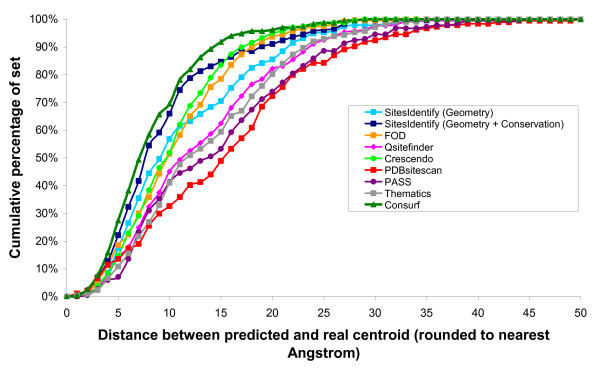
**Comparison of distances between the real centroid and the predicted centroid for each method**. The cumulative percentage of the set that have differences between the real and predicted active site centroids at each distance are shown for each method.

The prediction accuracies achieved for each method are shown in Table 2 and comparison of the distances between the predicted centroid and real centroid for each method are shown in Figure 5.

**Table 2:** Prediction accuracies achieved for each functional site prediction method.

Method	Absolute Recall Rate	Relative Recall Rate	Average Distance between Predicted and Real Centroid (Å)
SitesIdentify			
Uniform charge method	47.6%	63.0%	11.2
Conservation method	56.9%	74.7%	9.4
Consurf	58.6%	78.2%	8.2
Crescendo	46.9%	63.8%	10.3
FOD	39.7%	56.1%	10.6
QSiteFinder	40.1%	53.0%	13.0
PDBSiteScan	28.1%	38.4%	15.5
PASS	36.6%	49.3%	14.8
Thematics	35.8%	48.9%	13.5

The absolute and relative recall rates achieved along with the average distance between real and active site centroids for each method.

**Figure 6:**
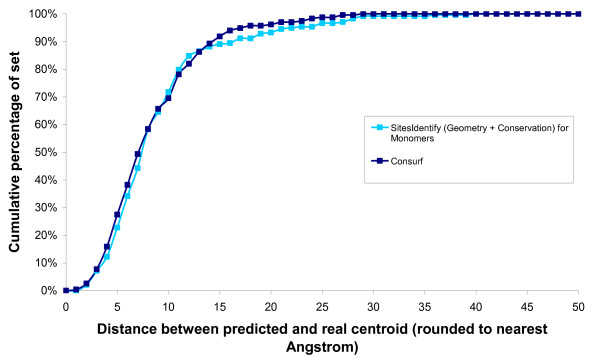
**Comparison of distances between the real centroid and the predicted centroid for Consurf and SitesIdentify run on monomer structures**. The cumulative percentage of the set that have differences between the real and predicted active site centroids at each distance are shown for both methods.

The conservation-based method of SitesIdentify achieved an average relative recall rate of 74.7%, which is comparable to that of the method with the highest accuracy, Consurf (78.1%). In order to extract site predictions for Consurf, all residues with a conservation score of 9 were assumed to be functional residues. For structures with more than one chain residue predictions were taken from the first chain only in order to avoid calculating the incorrect active site centroid from separate sites on multiple chains. Consurf was therefore effectively run on monomer structures rather than the true asymmetric unit. It is worth noting that when SitesIdentify is also run on monomer structures formed from only the first chain in the file it achieves a very similar performance to Consurf (see Figure 6).

Both Consurf and SitesIdentify are based around predicting conserved residues as functional site residues but whilst Consurf appears to perform slightly better overall, it could not produce predictions for three of the proteins in the set (1C3J, 1DMU and 1PGS) as it was unable to identify enough homologues. SitesIdentify uses both a combination of residue conservation information with an electrostatics-based cleft-finding algorithm and so it still gives predictions where there is little or no conservation information available. SitesIdentify was able to recall 100% of the annotated catalytic residues for the three proteins in this set for which Consurf did not make any prediction. SitesIdentify, therefore, is likely to give better predictions for structures from uncharacterised families, such as those being generated by structural genomics initiatives.

As discussed previously, residue conservation is known to be less indicative of functionality for non-enzymes than for enzymes[8,49,59], and here purely conservation-based approaches, such as Consurf and Crescendo, achieved a lower average recall rate compared to both SitesIdentify methods on a small set of non-enzymes (see Additional File 2).

PDBSiteScan achieved the lowest absolute and relative recall rates (28.1% and 38.4%, respectively) and also the largest average distance between predicted and real active-site centroids (15.5Å). PDBSiteScan scans the query protein against proteins of known annotation. In this analysis the test set consists of enzymes with known annotation and therefore it was necessary to reject predictions that simply accessed the annotation of any of these test proteins. As the number of proteins with well-characterised active site information is limited, removing these proteins from the set that PDBSiteScan compares to will obviously reduce the prediction power of the method. If tested on proteins outside of this set (i.e. proteins with uncharacterised functional sites) the prediction accuracy may increase.

Q-SiteFinder identifies energetically favourable methyl binding sites by calculating the interaction energy between the protein and a methyl probe and then ranking clusters of probes by their total interaction energy. Similar to the electrostatics-based method of SitesIdentify, Q-SiteFinder is essentially a cleft-finding algorithm. Despite similar approaches the uniform charge method of SitesIdentify achieves a 10% higher relative recall rate than Q-SiteFinder. Both Q-SiteFinder and SitesIdentify performed better than the other cleft-finding method, PASS, which also selects for cleft depth. Since SitesIdentify implicitly detects the atom density around a cleft rather than the cleft geometry itself, it suggests that this may be a contributing factor to the increased accuracy over PASS.

It is interesting that whilst SitesIdentify (charge-based) and Crescendo use very different approaches they give very similar accuracies on this dataset, suggesting that both conservation and geometrical information are equally useful in identifying functional sites. The combination of both of these approaches in the conservation-based method of SitesIdentify further improves the accuracy achieved by either one alone.

## Conclusion

Here we present a functional site prediction tool, SitesIdentify. We have shown that this tool compares favourably to other available functional site prediction tools in a comparison of methods on a non-redundant set of 237 enzymes with annotated active sites. The combination of structure-based and conservation-based approach in this tool produces accurate results, whilst a non-conservation based approach is also available for proteins that perhaps occupy remote fold-space and have no closely related homologues. Such methods are useful for identifying functional sites, and therefore informing about potential protein function, for structures of uncharacterised proteins.

## Availability and Requirements

**Project name: **SitesIdentify

**Project home page: **http://www.manchester.ac.uk/bioinformatics/sitesidentify/

**Operating system(s): **Platform independent

**Programming language: **PHP, Perl, Fortran, Jmol, Javascript.

**Other requirements: **e.g. Javascript enabled web browser

**License: **Free for all users

**Any restrictions to use by non-academics: **None

## Authors' contributions

TB carried out the comparison analysis, created the web-server application and wrote the manuscript, whilst JW supplied electrostatics code, PC and RG supplied conservation calculation code and PC and SB provided some website code. JW and AJD directed the design of the application and critically revised the manuscript. All authors read and approved the final version.

## Supplementary Material

Additional file 1 **SitesIdentify source code**. Compressed file containing the source code for SitesIdentify. Click here for file

Additional file 2 **Non-enzyme ligand binding comparison**. A table showing the prediction accuracies achieved for each functional site prediction method on 13 non-redundant non-enzyme structures with bound ligands from the Q-SiteFinder test set (Laurie and Jackson, 2005). Click here for file

Additional file 3 **PDB ID codes for the test dataset**. A list of all PDB ID codes for the structures used in the comparison test. Click here for file

Additional file 4 **Functional site prediction tools not included in the comparison analysis**. A list of the functional site prediction tools not used in the comparison analysis and the reason for their non-inclusion. Click here for file
