# Supplementary material for: SitesIdentify: a protein functional site prediction tool
Source: BMC Bioinformatics. 2009 Nov 18;10:379. doi: 10.1186/1471-2105-10-379 (PMC2783165; doi:10.1186/1471-2105-10-379)
Supplement: Additional file 2 — Non-enzyme ligand binding comparison. A table showing the prediction accuracies achieved for each functional site prediction method on 13 non-redundant non-enzyme structures with bound ligands from the Q-SiteFinder test set (Laurie and Jackson, 2005). [file 1471-2105-10-379-S2.doc]

| **Method** | | **Absolute Recall Rate** | **Relative Recall Rate** | **Average Distance between Predicted and Real Centroid (Å)** |
| --- | --- | --- | --- | --- |
| SitesIdentify | |  |  |  |
|  | Uniform charge method | 45.0% | 69.1% | 13.6 |
|  | Conservation method | 41.0% | 62.2% | 11.8 |
| Consurf | | 36.3% | 52.1% | 13.1 |
| Crescendo | | 44.2% | 65.8% | 11.8 |
| FOD | | 22.9% | 33.7% | 18.1 |
| QSiteFinder | | 33.6% | 54.0% | 12.5 |
| PDBSiteScan | | 11.4% | 23.5% | 19.5 |
| PASS | | 37.5% | 47.1% | 17.4 |
