# Supplementary material for: SitesIdentify: a protein functional site prediction tool
Source: BMC Bioinformatics. 2009 Nov 18;10:379. doi: 10.1186/1471-2105-10-379 (PMC2783165; doi:10.1186/1471-2105-10-379)
Supplement: Additional file 3 — PDB ID codes for the test dataset. A list of all PDB ID codes for the structures used in the comparison test. [file 1471-2105-10-379-S3.doc]

| 1ssx | 1h2r | 1dxe | 1gpr | 2plc | 1b65 |
| --- | --- | --- | --- | --- | --- |
| 1eb6 | 1gcu | 1c3c | 1f7l | 1wgi | 1al6 |
| 1p1x | 1g6t | 1bp2 | 1c3j | 1r16 | 1abr |
| 1qj4 | 1dw9 | 2jcw | 1bg0 | 1pa9 | 12as |
| 1qv0 | 2xis | 2acy | 1sox | 1oas | 1rbl |
| 1itx | 1ru4 | 1qrg | 1qcn | 1nsp | 1qd6 |
| 2nlr | 1d3g | 1qaz | 1gog | 1nid | 1pya |
| 1nww | 1uaq | 1e1a | 1fua | 1m9c | 1pfk |
| 1qtn | 1s95 | 1cg6 | 1d6o | 1gpj | 1nir |
| 1o9i | 1r6w | 1uro | 1d0s | 1eh6 | 1n20 |
| 1e7l | 1qq5 | 1tys | 1bs4 | 1e2a | 1nn4 |
| 2tps | 1moq | 1tml | 1b93 | 1bou | 1mvn |
| 2pth | 1lam | 1j79 | 1apy | 1a05 | 1mqw |
| 1vlb | 1jnr | 1foa | 1a4i | 2cpo | 1ef0 |
| 1qje | 1dl2 | 1chd | 1a2t | 1yve | 1cd5 |
| 1fy2 | 1cs1 | 1r51 | 1mrq | 1nml | 1r4f |
| 1dbf | 1aop | 1pgs | 1l1d | 1lci | 1q3q |
| 1nlu | 1v0y | 1p4n | 1kp2 | 1f75 | 1ndo |
| 1rhs | 1qgx | 1oe8 | 1jhf | 1bol | 1f8x |
| 1hdh | 1eug | 1lbu | 1jdw | 1aj0 | 1dhf |
| 2eng | 7odc | 1jh6 | 1i6p | 1otg | 1cev |
| 1c0k | 1uqr | 1j53 | 1chm | 1k4t | 3nos |
| 135l | 1qhf | 1j09 | 1akd | 1fro | 3mdd |
| 7atj | 1qd1 | 1g72 | 1oj4 | 1f2v | 2toh |
| 1pyl | 1p3d | 1dup | 1oac | 1dmu | 1d8h |
| 1nln | 1o04 | 1d4a | 1hxq | 1c2t | 1a79 |
| 1nf9 | 1gqg | 1cgk | 1vid | 1bwp | 1vas |
| 1dj0 | 1d1q | 2sqc | 1pii | 3eca | 1jms |
| 1oyg | 1ako | 1tph | 1js4 | 2ypn | 1dve |
| 1mla | 2bbk | 1pud | 1do6 | 1uam | 1dqa |
| 1m6k | 2apr | 1m21 | 1aug | 1lij | 1c9u |
| 1e19 | 2ahj | 1l1l | 1a95 | 1lba | 1b5t |
| 1qh5 | 1j7g | 1g24 | 1nhx | 1k82 | 1qpr |
| 1dci | 1i19 | 1daa | 1hrk | 1h3i | 1dco |
| 1p5d | 1dae | 1uf7 | 1g79 | 1ez1 | 1r76 |
| 1ah7 | 1rhc | 1ro7 | 1fgh | 1dqs | 1bt1 |
| 1snn | 3cla | 1mka | 1ecm | 1dnp | 1jm6 |
| 1l6p | 1mud | 1k30 | 1ecl | 1dio |  |
| 1hka | 1kaz | 1jfl | 1d8c | 1ca3 |  |
| 1hfe | 1fnb | 1ir3 | 1d2t | 1brw |  |
