# Supplementary material for: SitesIdentify: a protein functional site prediction tool
Source: BMC Bioinformatics. 2009 Nov 18;10:379. doi: 10.1186/1471-2105-10-379 (PMC2783165; doi:10.1186/1471-2105-10-379)
Supplement: Additional file 4 — Functional site prediction tools not included in the comparison analysis. A list of the functional site prediction tools not used in the comparison analysis and the reason for their non-inclusion. [file 1471-2105-10-379-S4.doc]

| **Name** | **Reference publication** | **Reason for non-inclusion in analysis** |
| --- | --- | --- |
| CrPred | Zhang et al., (2008) | Technical reasons. |
| CSS | Torrence et al. (2005) | Scans test set. |
| FrPred | Fischer et al. (2007) | Processing time. (could only process <500 residues) |
| Functional Site Prediction Server (FSPS) | Cheng et al. (2005) | Technical reasons. |
| MFS | Wang et al. (2008) | Technical reasons. |
| Par3D | Goyal et al. (2007) | Prior knowledge needed |
| PINTS | Stark and Russell (2003) | Technical reasons. |
| PinUP | Liang et al. (2006) | Processing time. |
| Protemot | Chang et al. (2006) | Cannot process results |
| PvSOAR | Binkowski et al. (2004) | Technical reasons. |
| SARIG | Amitai et al. (2004) | Technical reasons. |
| SiteEngine | Shulman-Peleg et al. (2005) | Prior knowledge needed |
| SPASM/RIGOR | Kleywegt (2005) | Prior knowledge needed |
| SuMo | Jambon et al. (2005) | Processing time. |

**Table: Functional site prediction tools not included in the comparison analysis.**

Reasons for non-inclusion in the analysis are further explained below:

***Technical reasons*.** Web-servers that produced errors on attempting to submit a protein or accessing results pages were not included.

***Prior knowledge needed.*** These prediction methods needed prior knowledge about the active site as they searched for similarity between the test protein and a user defined motif, pattern or protein.

***Cannot process results.*** The results are not given in a form that can be automatically processed (in this case the prediction was displayed as a graphic of the test protein with the catalytic residues highlighted).

***Processing time.*** Due to the large number of proteins used in the test set (237), the time taken to compute predictions is important to this analysis. A tool was excluded if results were not returned within 10 minutes for an example test protein of 330 residues (PDBID: 12as).

***Scans test set.***  CSS scans the CSA in order to compare the test protein to proteins with annotation in the CSA. It was deemed unsuitable for this analysis since the test set is derived from the CSA.
